# Two-Stage Total Hip Arthroplasty for Primary Advanced Septic Arthritis of the Hip in Adults

**DOI:** 10.1155/2022/8387465

**Published:** 2022-04-04

**Authors:** Zhenzhong Li, Congcong Wei, Xiangke Li, Mengxuan Yao, Huijie Li

**Affiliations:** ^1^Department of Orthopedics, Handan First Hospital, Handan, 056000 Hebei, China; ^2^Department of Orthopedics, The Third Hospital of Hebei Medical University, Shijiazhuang, 050000 Hebei, China

## Abstract

**Background:**

Comparing the outcomes of debridement and total hip arthroplasty (THA) with antibiotic-loaded spacer implantation and subsequent THA for the treatment of patients affected by primary advanced septic arthritis (SA) of the hip in adults.

**Methods:**

All of the 20 patients (20 hips) underwent two-stage surgery. Nine patients were submitted to surgical debridement first and then THA (group 1), while 11 patients were treated with antibiotic-loaded spacer and subsequent THA (group 2). Patients were evaluated based on the recurrence of infection, Harris hip score, visual analogue scale (VAS) pain score, and leg length discrepancy.

**Results:**

No cases of infection, deep vein thrombosis, death, and loosening of the hip prosthesis were observed during follow-up. The mean follow-up time was 29.09 ± 10.80 months in group 1 and 28.22 ± 14.80 months in group 2. Before the THA surgery, the mean leg length discrepancy was 2.80 ± 2.03 cm in group 1 and 0.50 ± 0.23 cm in group 2 (*P* < 0.05). In the latest follow-up, the Harris hip scores of patients were 90.33 ± 4.85 in group 1 and 94.36 ± 2.34 in group 2 (*P* < 0.05), respectively. There was no statistically significant difference in the VAS pain score of the hip between the two groups (*P* > 0.05).

**Conclusions:**

Debridement and antibiotic-loaded spacer and subsequent THA were effective in eradicating the infection for advanced SA. However, antibiotic-loaded spacer and subsequent THA was superior for effectively maintaining the length of the lower limb and function of the hip.

## 1. Introduction

Septic arthritis (SA) is a devastating disease in adults with high morbidity and mortality, which mainly affect the knee and hip joints [1, 2]. Ineffective treatment may lead to osteomyelitis, systemic sepsis, and leg length discrepancy of the hip [3]. Despite the surgical treatment, the occurrence of poor outcomes can still reach 22% [4]. Primary SA of the hip is defined as an infection of the hip joint that develops in the absence of a fracture, insertion of an implant or prosthesis, and surgical procedure. Until now, multiple treatment options are available, such as repeated aspirations, open arthrotomy, arthroscopic drainage, and two-stage total hip arthroplasty (THA) [5, 6]. However, treatment for advanced SA of the hip can be confronted with great challenges.

In the case of SA with bone involvement, aggressive surgery including joint resection and resection arthroplasty has been conducted. For example, a previous study by Girdlestone [7] has reported that resection arthroplasty is an effective treatment for SA of the hip. However, it may be associated with poor functional outcomes. The subsequent report has found that the use of antibiotic-loaded spacers in two-stage THA may be considered a suitable method for the treatment of primary SA in adults [8]. In addition, Li et al. [9] have reported that two-stage THA using debridement or femoral head resection with antibiotic-loaded spacer implantation is effective in the treatment of advanced active tuberculosis of the hip and has demonstrated that the outcomes of the two procedures are consistent. However, it is still not clear which of the two procedures has the advantage or disadvantage for the primary advanced SA patients. Therefore, it is necessary for us to further demonstrate the comparison of debridement with antibiotic-loaded spacer implantation in the first stage of two-stage THA in adults.

The primary aim of the present study was to assess the eradication of infection for primary SA of the hip between debridement and antibiotic-loaded spacer and subsequent THA. The secondary aim was to compare the differences of other indicators between the two surgeries including the Harris hip score, leg length, visual analogue scale (VAS) pain score, time between the two operations, blood loss, and operation duration. These findings of this study may provide a new treatment for primary advanced SA of hips in adults.

## 2. Material and Methods

### 2.1. Patients

We conducted a single-center, retrospective cohort study of patients treated with THA for SA of the hip. From January 2014 to December 2017, 21 patients with SA of the hip were eligible for enrollment in our study. Among them, 1 patient decided to leave the hospital and was treated in the local hospital after being diagnosed. A total of 20 patients were finally included. Inclusion criteria were patients who (1) were aged ≥18 years, (2) had a definite diagnosis of acute SA of the hip joint, (3) had advanced SA with destruction of hip joint, and (4) had a set of complete medical records (including nursing records) and preoperative and postoperative radiographs. Exclusion criteria were patients who (1) had early SA of the hip, (2) had multiple arthritis, (3) had incomplete medical records, (4) were not willing to be followed up, and (5) had an immune deficiency.

### 2.2. Patient Diagnosis

**Figure 1:**
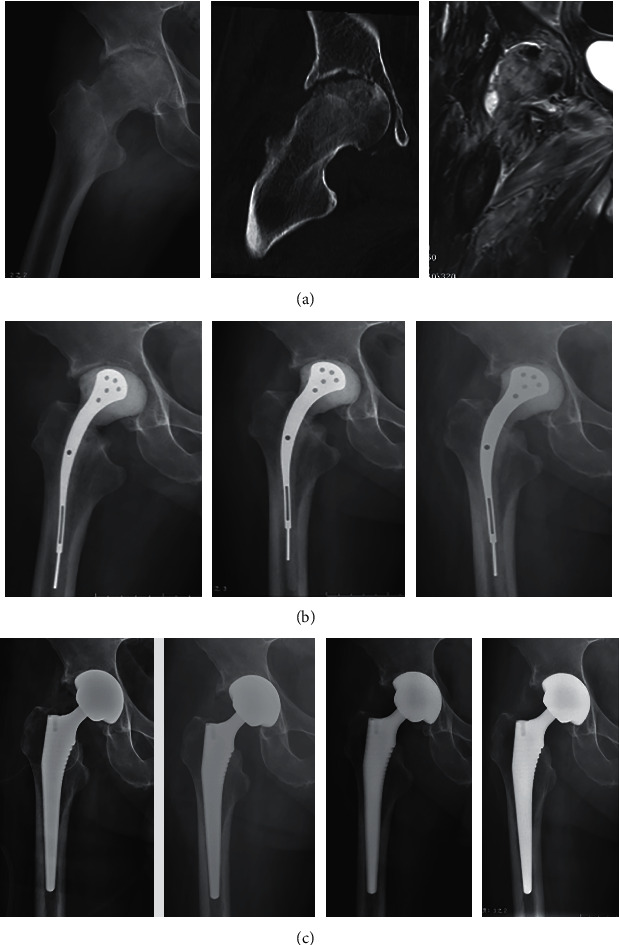
Radiographs of a 49-year-old man with pain in his right hip. (a) Anteroposterior X-ray and coronal CT images of the pelvis showed narrowing of hip joint space and destruction of bone. Coronal MRI (cut) of the pelvis showed diffuse edema of the right hip joint involving almost all muscles of the right hip. (b) The femoral head was excised and implanted with an antibiotic cement prosthesis: 5 days, 1 month, and 3 months after the operation, respectively. (c) The postoperative imaging findings of hip replacement at 0 days, 1 month, 3 months, and 19 months, respectively.

**Figure 2:**
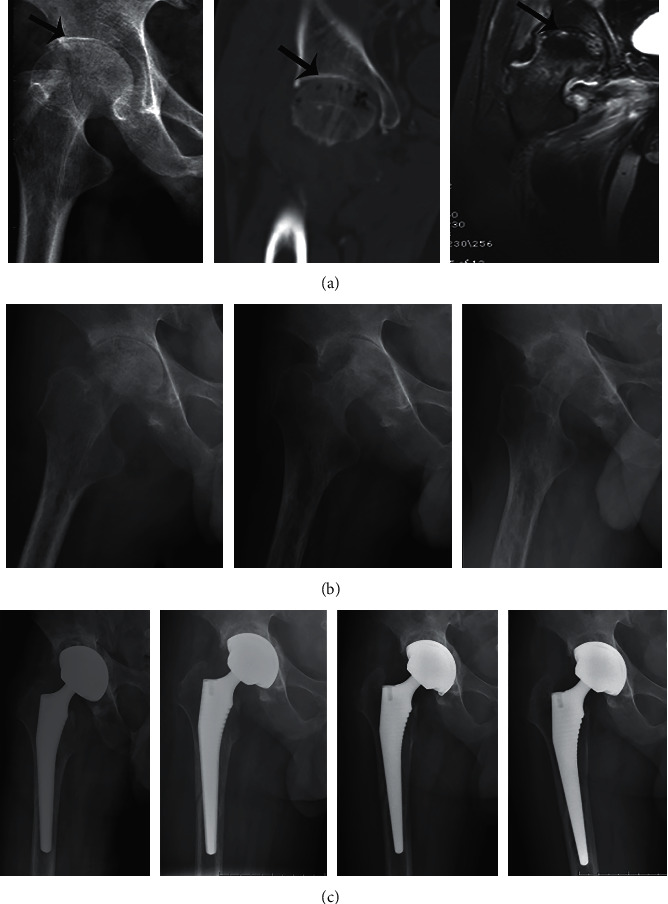
Radiographs of a 43-year-old man with pain in his right hip joint. (a) Anteroposterior X-ray and coronal CT images of the pelvis showed that the space of the hip joint became narrow and articular cartilage disappeared (arrow). Coronal MRI of the pelvis showed diffuse edema of the right femoral head. (b) X-ray examination of the hip joint at 4 months, 12 months, and 15 months after debridement operation. (c) The postoperative images of hip replacement at 7 days, 2 months, 5 months, and 15 months, respectively.

**Figure 3:**
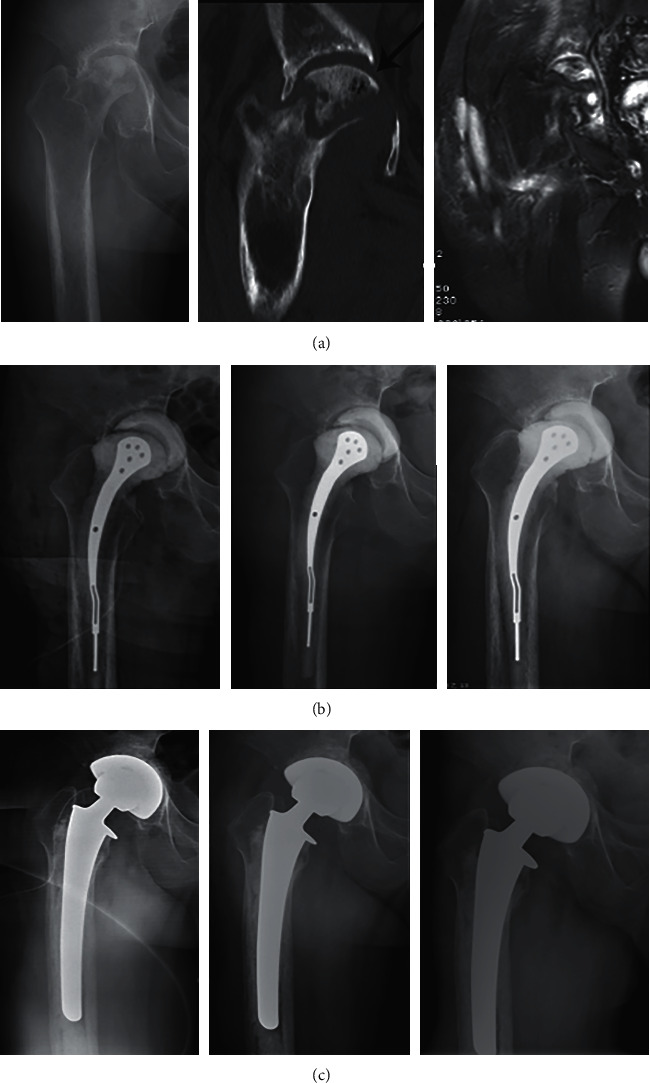
Radiographs of a 54-year-old man with right hip joint pain and severe limitation of movement. (a) Anteroposterior X-ray and coronal CT images of the pelvis showed that the right acetabulum was invaginated and the acetabulum wall was damaged (arrow). Coronal MRI of the pelvis showed inflammatory changes in the right hip joint. (b) The postoperative imaging of femoral head resection with antibiotic cement implantation at the same day, 1 month, and 6 months, respectively. (c) The imaging findings after THA at 0 days, 1 month, and 12 months, respectively.

Patients with SA of the hip had clinical symptoms of infection including pain, sudden chills and fever, local swelling, and limited range of motion. In addition, complete blood cell count (CBC), C-reactive protein (CRP), and erythrocyte sedimentation rate (ESR) were measured. X-ray, CT, and MRI were also performed. X-ray showed narrowing of the hip joint space for advanced SA of the hip. CT could detect local edema, osteonecrosis, osteomyelitis, and hip bone destruction. MRI provided good resolution for the detection of joint effusion, bone differentiation, and soft tissue infection. MRI findings of SA patients included joint effusion, cartilage and bone destruction, soft tissue abscess, and bone edema (Figures 1–3) [10]. The diagnosis of SA synthesized the results of these tests. If the patient was suspected of having septic arthritis, the PPD test, Brucella agglutination test, and needle aspiration of the hip were then added. The preoperative samples of 6 cases were positive, including Staphylococcus aureus in 3 cases, Brucella in 1 case, Mycobacterium tuberculosis in 1 case, and Klebsiella pneumoniae in 1 case. The advantages and disadvantages of two-stage THA were addressed for a specific patient, and then, a surgical plan was developed according to the patient's willing [11].

### 2.3. Surgical Technique

All of the 20 patients (20 hips) underwent two-stage surgery. Nine patients were submitted to surgical debridement first and then THA (group 1), while 11 patients were treated with antibiotic-loaded spacer and subsequent THA (group 2). All operations were performed by four orthopedic surgeons in our department.

During the first stage, debridement was performed in group 1, and femoral head resection with antibiotic-loaded spacer implantation was performed in group 2. The posterolateral approach was performed in all patients. The inflamed soft tissues, necrotic bones, abscesses, and sinus tracts were totally debrided in the two groups during the first stage. Then, the 4-5 samples were taken from different parts of the suspicious joint for pathological examination and bacterial culture. For patients undergoing debridement, an irrigation tube was placed in operation and continued to be rinsed with gentamicin solution after the operation. For patients undergoing femoral head resection and antibiotic-loaded spacer implantation, the femoral head was removed and a cement spacer containing gentamicin was installed (Figure 1). Until the patient's symptoms disappeared and the serum became negative, the patient was allowed to be discharged and was required to take oral antibiotics for at least two weeks [10, 11]. CBC, CRP, and ESR were detected for weekly regular monitoring. In general, traction for the affected limb was conducted if the pain (VAS score of 4-6) affected sleep after the operation. Affected limbs were strengthened by functional exercise at postoperative 48 h [10, 12].

During the second stage, all procedures were performed via the posterolateral approach, with the patient in a lateral position. When the joint was opened, no pus and no inflammatory edema of soft tissue around the joint were confirmed under the naked eye. Then, the hypertrophic soft tissue and scar were removed as thoroughly as possible in the direct visualization. Iodophor and hydrogen peroxide were used to immerse the hip joint cavity to reduce the probability of infection. The acetabular wall defect in 2 cases was restored by the Titanium Acetabular Graft Cup, and then, acetabular impactors were used to perform impaction grafting. The gap between cup and host bone was completely filled by allogeneic morselized bone. On completion of cage placement and bone grafts, an acetabular polyethylene liner was fixed with high radiopaque bone cement containing gentamicin. In addition, 3 patients with central acetabular defect underwent impaction grafting using acetabular impactors, and then, a larger acetabular cup was implanted. Besides, 1 patient had a high hip dislocation secondary to SA of the hip. Structural autograft obtained from the femoral neck was implanted on the patient's upper acetabular bone and fixed with screws. The remaining 14 patients were installed with matching acetabular cup after grinding of the acetabulum. The usage of antibiotics depended on the susceptibility of the first-stage organism identified in joint fluid culture. Antibiotics were chosen empirically if no organism was identified.

### 2.4. Data Collection

The scanning edition of electronic medical records was reviewed in detail to retrieve pertinent information, including demographic data (gender, age, height, and weight), the preoperative and postoperative clinical evaluation (CBC, CRP, and ESR), imaging evaluation (X-ray, CT, and MRI), Harris hip score, and VAS pain score. Clinical data were all recorded during follow-up.

### 2.5. Follow-Up

Anteroposterior and lateral radiographs of the hip, full-length view of the lower extremities, CT, and MRI of the hip were taken for the preoperation. In our department, patients were required to have a regular follow-up in the 1st month, the 3rd month, the 6th month, and the 12th month after the operation. Anteroposterior and lateral radiographs of the hip were taken at each follow-up. All of the 20 patients had adequate preoperative and postoperative imaging examinations.

### 2.6. Statistical Analysis

All data were performed using SPSS 19.0 software (IBM, Armonk, NY, USA). Quantitative data were described using means ± standard deviation (SD). The Mann–Whitney *U* test was used to compare continuous categorical variables, including the Harris hip score and VAS pain score between the two groups. A *P* value of less than 0.05 was considered statistically significant.

## 3. Results

All of the 20 patients had a postoperative follow-up. The mean age was 50.60 ± 10.82 years. There were no significant differences in laboratory indexes, Harris hip scores, VAS pain scores, and body mass index (BMI) between the two groups preoperatively (Table 1).

In the first stage, the results of bacterial diagnosis in 20 patients were Staphylococcus aureus (4 cases), Brucella (1 case), Mycobacterium tuberculosis (1 case), and Klebsiella pneumoniae (1 case), and the rest were negative. In the first stage of surgery, the two groups had similar operation duration and blood loss (Table 2). The mean follow-up time was 28.22 ± 14.80 months in group 1 and 29.09 ± 10.80 months in group 2, respectively. No recurrence of SA occurred after the second stage of the two-stage THA. No loosening or dislocation of prosthesis, thrombosis, or death was found in all patients during the follow-up period. The symptoms of the patients were greatly improved. The Harris hip score increased in both groups after two-stage THA surgery (28.45 ± 3.86, 29.56 ± 4.00 vs. 94.36 ± 2.34, 90.33 ± 4.85), and the VAS pain score decreased after two-stage THA surgery (5.36 ± 1.12, 4.67 ± 0.71 vs. 0.09 ± 0.30, 0.44 ± 0.53).

**Figure 4:**
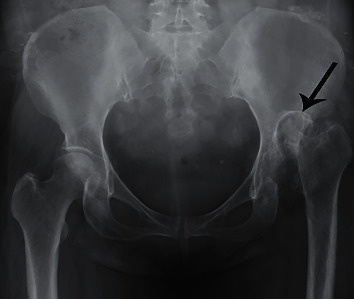
Anteroposterior X-ray images of the pelvis showed that the left femoral head had been absorbed, and the rest of the femoral neck had been dislocated and formed a pseudarthrosis (arrow).

We also observed many differences between the two methods in Table 2. For the first stage, the mean duration of postoperative hospitalization in group 1 (19.00 ± 4.95 days) was significantly longer than that in group 2 (12.09 ± 2.17 days) (*P* = 0.001). The patients had a better recovery of hip joint function in group 2. Although the patient in group 1 underwent debridement, the bones forming the hip joint may continue to be damaged and even caused a high dislocation of the hip joint (Figure 4). The mean Harris hip score in group 1 was lower than that in group 2 after the first stage of two-stage THA (all *P* < 0.05). The interval between the two stages of surgery in group 1 (10.30 ± 8.70 months) was longer than that in group 2 (3.43 ± 1.50 months) (*P* = 0.039). Before the THA surgery, the mean leg length discrepancy was 2.80 ± 2.03 cm in group 1 and 0.50 ± 0.23 cm in group 2 (*P* < 0.05). In the second stage, the operation time of group 1 (175.00 ± 68.74 min) was significantly longer than that of group 2 (131.36 ± 30.67 min) (*P* = 0.047).

For the latest follow-up, the Harris hip score was 90.33 ± 4.85 points in group 1 and 94.36 ± 2.34 points in group 2 (*P* = 0.027). There were no significant differences in the mean VAS score between group 1 (0.44 ± 0.53) and group 2 (0.09 ± 0.30) (*P* = 0.077), but 4 of the 9 patients (44.44%) in group 1 had occasional hip discomfort or mild aches, compared with 1 of the 11 patients (9.10%) in group 2 (Figure 3) (Table 2).

## 4. Discussion

Primary SA of the hip is rare in adults, which leads to serious complications and significant morbidity and mortality if it is not diagnosed and treated promptly [13, 14]. Two-step total hip arthroplasty has been used when the hip suffers from severe destruction [15]. In our study, we found that the main type of microorganism was Staphylococcus aureus. Furthermore, we compared the effect of treatment between debridement and femoral head resection with antiloaded spacer implantation in the first stage of surgery. The findings showed that antibiotic-loaded spacer implantation and subsequent THA was superior to debridement and then THA with regard to mean leg length discrepancy of patients and Harris hip scores.

Previous studies have revealed that the causative microorganism is closely related to SA of the hip [16, 17]. In our cases which were diagnosed with a specific bacterial infection, Staphylococcus aureus accounted for 57.14%. In general, bacterial cultures have a high false-negative rate. For example, a previous report has reviewed 133 articles and has found that the positive rate of bacterial culture in synovial fluid is almost 40%-50% [18]. Our positive culture rate is about 25% (5 out of 20 in the present series) in the intraoperative diagnosis of bacterial culture. We speculated that the reason for the low rate may be related to the preoperatory antibiotic therapy, fastidious microorganisms, sampling technique, and laboratory procedures of culture bacteria [19]. In addition, using antibiotics might control joint infection as well as prevent further septic metastases [20].

Generally, infection control and restoring hip function were two tasks for physicians when treating SA of the hip. Therefore, surgery and antibiotics are key factors for the treatment of primary SA of the hip. Previous studies have demonstrated that two-stage THA is considered a suitable method for the treatment of primary SA. For example, Bauer et al. [21] have concluded that two-stage THA and one-stage THA are successful in almost 90% of patients with SA and effective for eradication of the infection. A similar study has also demonstrated that intervals between spacer implantation and subsequent THA can effectively control the rate of infection [20]. In our study, debridement or antibiotic-loaded spacer implantation and subsequent THA achieved the goal of eradication of the infection. In general, we identified that the indicators of complete infection control were the leukocytes, neutrophils, ESR, and CRP within the normal range and no effusion and edema in the hip joint after 2 weeks of drug withdrawal. The current study showed that the speed of serum in group 1 returning to normal was slower than that in group 2. It probably was because the patients in group 1 did not remove the femoral head during the first stage of THA operation, which caused the joint cavity to not be completely cleaned. The reason was conformed to a previous study, which had reported that residual focus might exist due to destruction of the joint or the existence of sinus tracts though no macroscopic inflamed tissues were seen during operation [9]. Moreover, Li et al. [22] have reported that the use of antibiotic-loaded spacer implantation increases the local antibiotic concentration in the hip joint, and thus, the patient's inflammation is quickly and effectively controlled. Therefore, the interval between the two stages of surgery in group 1 was longer than that in group 2.

As we know, leg length discrepancy is an important factor for functional recovery of hip joints after THA [23]. Yoon et al. [24] have reported that femoral head resection with antibiotic-loaded spacer and subsequent THA could reduce the mean leg length discrepancy from 2.95 to 0.8 cm at postoperation. In our study, the mean leg length discrepancy was 2.80 ± 2.03 cm in group 1 and 0.50 ± 0.23 cm in group 2 before the THA surgery. Therefore, THA surgery in group 1 may be more difficult than that in group 2. Consistently, our study showed that the time of second-stage operation in group 1 was longer than that in group 2. This may be related to severe limb contracture around the hip joint in group 1. So, patients in group 1 needed soft tissue release to extend the length of the lower limbs, and the damaged acetabulum required bone grafting in the THA surgery. In terms of blood loss, a previous study has reported that the mean blood loss was almost 400 ml in THA surgery [6], which was consistent with our results in group 2. The current study showed that there was a little more blood loss in group 1; however, it was not significantly different from group 2. In general, the longer operation time represents the greater difficulty of operation, indicating the slower recovery of hip function [25, 26]. The abovementioned results suggested that operation time is closely associated with the recovery of hip function.

The most relevant clinical advantage of using an antibiotic-loaded spacer is that it helps maintain joint space and minimize the risk of large limb shortening [27, 28]. Furthermore, Rissanen et al. [29] have demonstrated that THA relieves pain and improves the walking ability of patients. Subsequent study has revealed that two-stage THA using interval spacer can improve the Harris hip score. In brief, no complications associated with the use of this novel spacer were found. The average Harris hip score improved from 35.2 preoperatively to 61.6 between the 2 stages to 93.6 in the latest follow-up [30]. In addition, two-stage THA using debridement or antibiotic-loaded spacer implantation can treat effectively patients with advanced active tuberculosis of the hip, and there were no significant differences between the two procedures [9]. However, the Harris hip score of patients who underwent antibiotic-loaded spacer implantation and subsequent THA was superior to that of patients who underwent debridement and then THA in our study. The difference in the Harris hip score was consistent with the result of operation difficulty and recovery time between the two groups. Compared with findings reported by Li et al. [9], the Harris hip score was higher in the case of short follow-up time, which may be related to relatively light destruction of bone and surrounding soft tissue of hip joints. Besides, the VAS pain score of the hip had no significant difference. The abovementioned findings suggested that antibiotic-loaded spacer implantation and subsequent THA was superior for operation time, leg length discrepancy, and Harris hip score.

There were several limitations to this study. First, this study was retrospective and conducted in a single-center medical institution with a small sample. Those abovementioned situations may result in sampling error, and conclusions may be influenced by unrelated factors. Second, the follow-up time was short. Additionally, most patients in group 1 had atrophy and contracture, and then, the soft tissue around the hip joint needed to be loosened before the second stage of THA operation in general. Therefore, it is important to perform the THA surgery as soon as possible in order to reduce bone and muscle atrophy. In the future, increased sample size and extended follow-up time should be carried out.

## 5. Conclusion

The present study suggested that both treatments were effective in eradicating the infection; however, antibiotic-loaded spacer implantation and subsequent THA was superior for effectively maintaining the length of lower limbs and function of hips. Therefore, treatment with antibiotic-loaded spacer and subsequent THA may be a promising option for the treatment of advanced primary SA of the hip in adults.

## Figures and Tables

**Table 1:** Baseline characteristics in two groups.

	Group 1 (*n* = 9)	Group 2 (*n* = 11)	*P* value
Age (years)	47.89 ± 10.34	52.82 ± 11.17	0.270
CRP (mg/l)	48.44 ± 45.58	67.41 ± 69.39	0.569
ESR (mm/h)	68.6 ± 31.23	56.36 ± 23.56	0.323
Leukocyte count (10^9^/l)	8.30 ± 2.06	7.26 ± 1.53	0.271
Neutrophil count (%)	67.68 ± 5.10	64.30 ± 12.20	0.621
BMI (kg/m^2^)	23.05 ± 3.49	23.57 ± 3.13	0.595
Harris hip score	29.56 ± 4.00	28.45 ± 3.86	0.541
VAS pain score	4.67 ± 0.71	5.36 ± 1.12	0.151
Time of symptoms preoperation (day)	44.22 ± 24.45	99.55 ± 81.53	0.101

CRP: C-reactive protein; ESR: erythrocyte sedimentation rate; BMI: body mass index; VAS: visual analogue scale.

**Table 2:** Comparison of perioperative outcomes in two groups.

	Group 1 (*n* = 9)	Group 2 (*n* = 11)	*P* value
PODFSH (day)	19.00 ± 4.95	12.09 ± 2.17	0.001^∗^
IBTSOS (months)	10.30 ± 8.70	3.43 ± 1.50	0.039^∗^
BMI (kg/m^2^)			
First stage	23.05 ± 3.49	23.57 ± 3.13	0.595
Second stage	25.39 ± 4.46	25.13 ± 2.87	0.569
Duration of operation (min)			
First stage operation	137.22 ± 39.06	130 ± 31.06	0.879
Second stage operation	175.00 ± 68.74	131.36 ± 30.67	0.047^∗^
Blood loss (ml)			
First stage operation	438.89 ± 119.32	595.45 ± 322.84	0.591
Second stage operation	1211.11 ± 1842.85	754.55 ± 697.66	0.697
Harris hip score			
First stage preoperation	29.56 ± 4.00	28.45 ± 3.86	0.541
First stage out of hospital	61.67 ± 3.04	73.55 ± 4.16	0.000^∗^
Second stage preoperation	74.44 ± 3.40	80.64 ± 2.66	0.001^∗^
Second stage out of hospital	81.78 ± 2.22	84.18 ± 2.14	0.049^∗^
Latest follow-up	90.33 ± 4.85	94.36 ± 2.34	0.027^∗^
VAS pain score			
First stage preoperation	4.67 ± 0.71	5.36 ± 1.12	0.151
First stage out of hospital	1.44 ± 0.53	1.36 ± 0.51	0.721
Second stage preoperation	1.67 ± 0.71	1.18 ± 0.41	0.077
Latest follow-up	0.44 ± 0.53	0.09 ± 0.30	0.077
Length discrepancy	2.80 ± 2.03	0.50 ± 0.23	0.000^∗^
Follow-up (month)	28.22 ± 14.80	29.09 ± 10.80	0.518

PODFSH: postoperative days of first stage hospitalization; IBTSOS: interval between the two stages of surgery; BMI: body mass index; VAS: visual analogue scale. ^∗^*P* < 0.05 (vs. group 1).

## Data Availability

All data generated or analyzed during this study are included in this published article.

## Abbreviations

SA: Septic arthritis
THA: Total hip arthroplasty
VAS: Visual analogue scale
CBC: Complete blood cell count
CRP: C-reactive protein
ESR: Erythrocyte sedimentation rate.
